# Digital Educational Strategies to Implement Evidence-Based Care for Atherosclerotic Cardiovascular Disease

**DOI:** 10.1007/s11883-026-01402-6

**Published:** 2026-03-16

**Authors:** Aileen Zeng, Carissa Bonner, Clara K Chow, Myron A Godinho, Liliana Laranjo, Brooke Nickel, Sarah Zaman, Edel O’Hagan

**Affiliations:** 1 Westmead Applied Research Centre, Faculty of Medicine and Health, University of Sydney, Sydney, New South Wales Australia; 2 The Leeder Centre for Health Policy, Economics and Data, Sydney Health Literacy Lab, School of Public Health, University of Sydney, Sydney, New South Wales Australia; 3 Department of Cardiology, Westmead Hospital, Sydney, New South Wales Australia; 4 Sydney School of Public Health, University of Sydney, Sydney, New South Wales Australia

**Keywords:** Health literacy, Text messaging, Conversational agents, Video education, Generative AI, Misinformation

## Abstract

**Purpose of Review:**

The Lancet Commission reconceptualises coronary artery disease as atherosclerotic coronary artery disease (ACAD)—a lifelong, systemic condition driven by atheroma rather than its late ischaemic consequences. This review summarises digital educational approaches that can support evidence-based ACAD prevention and management earlier in the disease course.

**Recent Findings:**

Evidence is strongest for text messaging, which improves medication adherence and smoking cessation and yields modest cardiometabolic benefits. Video education consistently enhances knowledge and engagement, though behavioural and clinical effects are mixed. Multicomponent and app-based strategies can support physical activity, weight, and glycaemic control, but results depend on design and integration. Conversational agents show small to moderate lifestyle improvements, mainly in primary prevention, but robust ACAD-specific outcome data are limited. Generative AI may enhance readability and access, yet clinical benefit is unproven and requires strong governance.

**Summary:**

Digital education can support ACAD care when well-designed, clinically integrated, and health-literacy aligned.

## Introduction

The Lancet Commission on rethinking coronary artery disease urges a shift from late-stage, ischaemia-focused care to atheroma-centred, lifelong prevention. Atherosclerosis begins early in life and progresses silently over decades. Reframing coronary artery disease as atherosclerotic coronary artery disease (ACAD) highlights opportunities for much earlier detection and sustained, lifelong prevention [1]. If behavioural and metabolic risk factors were eliminated or effectively controlled early, the global ACAD burden would fall dramatically. Modelling from the Commission estimates that eliminating major risk factors by 2050 could reduce ACAD deaths by 82.1% (approximately 8.7 million lives annually), underscoring the imperative to shift public health, health systems, and clinical practice towards earlier, sustained prevention [1].

**Fig. 1:**
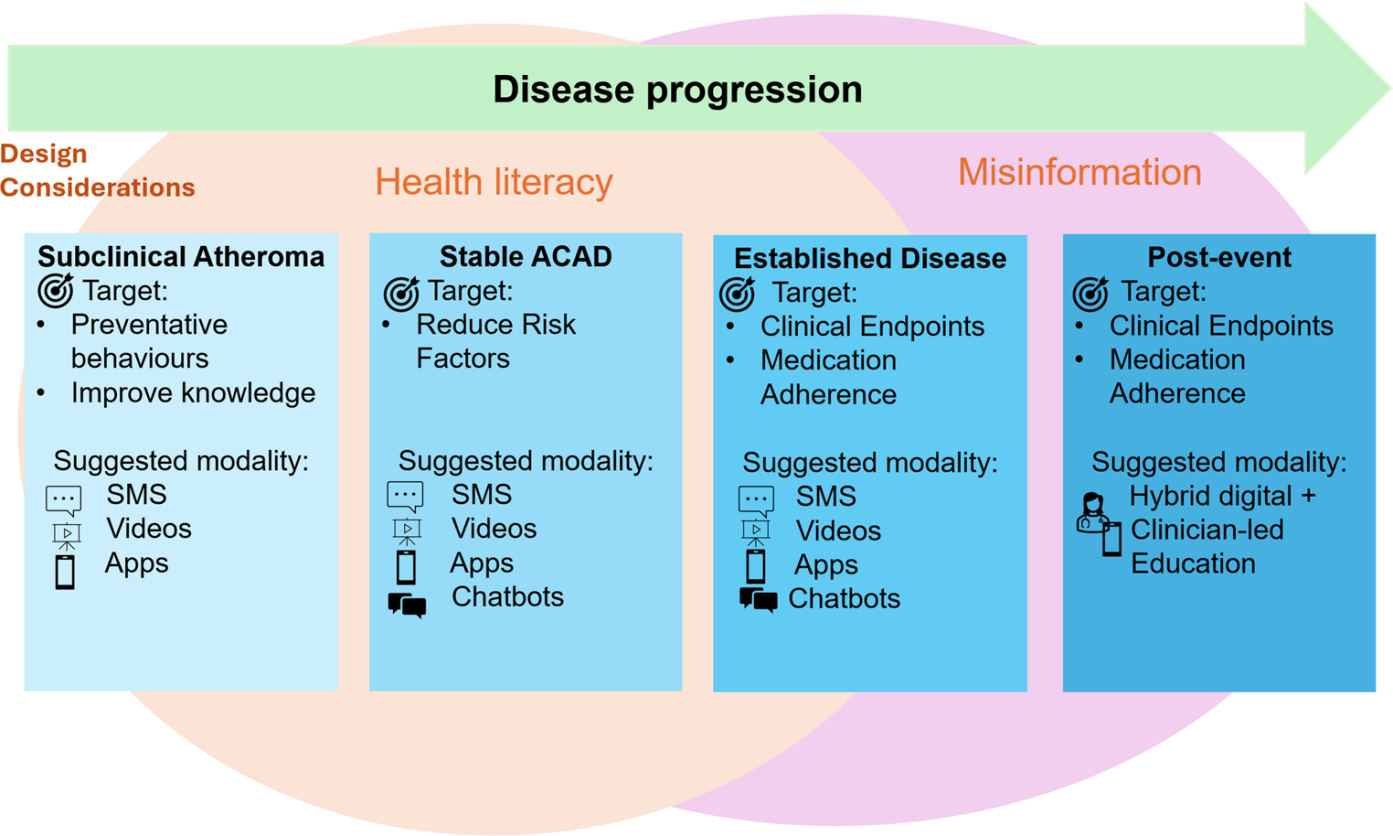
Overview of digital educational modalities intended for patients with the greatest relevance to ACAD prevention and management

The drivers of atherosclerosis include hypertension, dyslipidaemia, diabetes mellitus, smoking, poor diet, and low physical activity. These risk factors are common, cumulative, and behaviourally mediated. Scalable digital strategies are essential to achieve population impact. Educational interventions, historically delivered face-to-face, improve blood pressure, lipids, glucose, weight, diet, smoking and physical activity. Digital educational approaches are attractive for extending reach, enabling tailored support for long-term behaviour change, and reinforcing evidence-based care [2, 3]. This review examines digital educational modalities intended for patients with the greatest relevance to ACAD prevention and management including text messaging, video-based education, and multicomponent/app-based interventions, conversational agents, generative artificial intelligence (AI) and addresses health literacy and misinformation as cross-cutting determinants of effectiveness (Fig. 1). We conducted a search of recent peer-reviewed literature in PubMed using keywords relevant to atherosclerosis and the themes of this review. We included English-language original studies and reviews directly addressing education delivery using digital health. We excluded non–peer-reviewed materials, opinion pieces, and articles lacking relevance to the review focus. Articles were selected based on relevance, recency, and contribution to current understanding.

### Text Messaging (SMS and Instant Messaging)

Text messaging have long offered a well-tested, scalable approach for delivering low-cost prompts and tailored micro-lessons via short message service (SMS) to improve ACAD risk factors [4–6]. SMS programs are typically one-way/two-way messaging services focused on information delivery rather than interactive dialogue. Education-oriented programs are often broad in remit, ranging from health promotion or reminder messaging, to providing personalised information [7]. These can be used to raise awareness, increase knowledge, reinforce medication adherence and promote behaviour change – such as improving diet, physical activity, and smoking cessation, to reduce both behavioural and metabolic risk factors [8].

Previous meta-analyses have demonstrated effects across multiple risk domains [9–12]. For weight and body mass index, regular behaviour change technique–based messages were associated with a mean change in BMI of − 0.43 kg/m² (95% CI − 0.63 to − 0.23) [9]. For physical activity, a meta-analysis of 13 studies (*n* = 1,346) reported increases in objectively measured steps per day with a small-to-moderate effect size of d = 0.38 (95% CI 0.19–0.58), however effects on moderate-to-vigorous physical activity were smaller and sometimes non-significant [10]. Instant messaging interventions (i.e. messaging delivered via the internet rather than a mobile phone network, such as WhatsApp) demonstrated moderate improvements in physical activity (Standardised Mean Difference(SMD) 0.52, 95% CI 0.21–0.83), and higher odds of smoking cessation (Odds Ratio (OR) 1.88, 95% CI 1.28–2.70) [11]. Evidence for smoking cessation strongly supports integration of texting into prevention pathways: a recent meta-analysis found participants receiving cessation texts were nearly twice as likely to quit as those receiving usual care (Risk Ratio (RR) 1.87, 95% CI 1.52–2.29), with additive benefits alongside pharmacotherapy in some trials [12], and complementary findings from instant-messaging interventions [11].

In hypertension, text messaging has produced clinically relevant reductions in systolic (approximately − 6 mmHg) and diastolic blood pressure (approximately − 2.7 mmHg), alongside moderate improvements in medication adherence (mean difference(MD) 0.62; 95% CI 0.37–0.86), though effects on lipids and BMI are inconsistent [7, 13, 14]. Meta-analyses of SMS-based diabetes interventions show modest but statistically significant reductions in HbA1c, fasting and post-prandial glucose, and systolic blood pressure [15–17]. Yet, meta-analysis of instant messaging show no difference on HbA1c (SMD = − 0.45, 95% CI [− 1.38, 0.48], *p* < 0.001), BMI (SMD = − 0.1, 95% CI [− 0.3, 0.1], *p* = 0.16) and haemoglobin (SMD = 0.32, 95% CI [− 0.29, 0.93], *p* = 0.04) outcomes [11]. In adults with type 2 diabetes, messaging alone may not significantly improve physical activity or anthropometrics, suggesting augmentation rather than replacement of comprehensive programs in this group [18].

From an implementation perspective, umbrella and Cochrane reviews emphasise that mobile phone text messaging is highly accessible, relatively low cost [19], and acceptable to patients, but the certainty of evidence for some clinical endpoints remains limited because of risk of bias and outcome inconsistency [16, 20, 21]. Engagement can decline due to message fatigue, and effects vary across cultural contexts. Equity considerations are critical: low digital and health literacy and limited access to mobile technology may attenuate benefits in underserved populations [22]. Overall, these findings suggest that texting should be embedded as a supportive tool within multifaceted implementation strategies, such as combining automated, tailored messaging with clinician counselling, pharmacotherapy, and self-monitoring, to support reduction of metabolic and behavioural risk factors for ACAD.

### Video-Based Education

Short, professionally developed videos delivered via clinical platforms or social media can convey complex information in accessible, culturally adaptable formats, supporting patients across a range of languages and literacy levels. Across conditions, video education consistently improves knowledge, although effects on behaviour and clinical outcomes are more variable [23–25].

A systematic review of 59 studies (*N* = 9,789) across chronic conditions including hypertension and diabetes found knowledge gains in 75% of the trials (30/40) and improved medication use in 52% (15/29). Approximately half of studies also reported improvements in health behaviours and self-efficacy, while effects on disease severity and health care utilisation were mixed [26]. The included trials also provide a mixed picture when reporting on condition-specific outcomes: in diabetes, some trials reported short-term reductions in HbA1c (3–6 months) that were not sustained at 6–12 months; and blood pressure generally showed little change, although one larger trial demonstrated improvements in LDL cholesterol [26].

Using evidence from people with established cardiovascular disease, including those with atrial fibrillation, a randomised clinical trial (RCT) demonstrated that clinician-led, video-based AF education significantly improved AF-specific knowledge at 90 days (OR 1.23; 95% CI 1.01–1.49), with even greater gains among participants who accessed the videos repeatedly (OR 1.46; 95% CI 1.14–1.88). This indicated that video education can enhance disease understanding in secondary prevention populations [27].

A key advantage of video-delivered education is the ability to provide culturally tailored messaging. For example, among African American adults with uncontrolled blood pressure, peer-led video narratives achieved a mean systolic reduction of 11.21 mmHg (95% CI 2.51–19.9) at three months following peer-led video narratives [28].

In healthier populations, video-based interventions appear to have promising effects on encouraging health lifestyle behaviours. Across 119 RCTs involving 118,935 participants, video counselling was associated with 7-day point-prevalence abstinence from smoking compared with brief advice [29]. Meta-analysis of 11 studies showed that video-delivered exercise programmes produced significant improvements in strength, balance, and mobility in older adults [30].

Given the limited evidence base, and the substantial methodological weaknesses, including heterogeneity in how videos were produced, who delivered them, and the roles of actors or narrators, it remains unclear whether observed effects are attributable to the video format itself or to the specific content and tailoring used in individual trials. As a result, the overall potential for video-based education to improve ACAD prevention and management remains uncertain.

When implemented, however, studies indicate that video-based education is feasible in both inpatient and outpatient settings, with high satisfaction and engagement [31–33]. Collectively these findings suggest video-based education is dependable for knowledge gain and engagement, but its behavioural and clinical effects remain heterogeneous, underscoring the need for rigorous, longer-term trials focused on ACAD outcomes including lipids, blood pressure, and adherence to lifestyle change.

### Multicomponent and App-Based Interventions

Smartphone apps frequently integrate educational content, self-monitoring tools, reminders, and increasingly conversational AI. Evidence from diabetes-prevention trials shows modest improvements in weight (MD − 1.85 kg) and BMI (− 0.90 kg/m²), with no meaningful effects or HbA1c and waist circumference [34]. Apps and fitness trackers have also been shown to increase physical activity in a clinically significant manner [1850 steps per day (95% CI 1247 to 2457)] in a meta-analysis of 28 RCTs, with interventions that added text-messaging and personalisation features being significantly more effective [35]. Recent trials of stand-alone blood pressure apps have shown mixed results: self-monitoring via connected devices did not outperform standard self-monitoring when behaviour change and medication support were minimal; lifestyle-support apps achieved small systolic reductions (~ 2 mmHg) in non-medicated patients; adding conversational AI to self-monitoring did not further reduce blood pressure [36–38]. In diabetes, a meta-analysis of 17 RCTs (*n* ≈ 2,946) reported modest but significant reductions in HbA1c (–0.38%), fasting glucose (–14.1 mg/dL), systolic blood pressure (–1.3 mmHg), LDL cholesterol (–6.1 mg/dL), and triglycerides (–6.5 mg/dL), alongside increased physical activity participation with apps [39].

Apps can also support smoking cessation: when combined with standard interventions or pharmacotherapy smartphone apps improve quit rates at 6 months (RR up to 2.85), and personalised behaviour therapy-based apps outperform traditional behavioural apps for short-term abstinence [40]. Evidence for weight and activity focused apps is similarly encouraging. A meta-analysis of 11 RCTs (*n* = 1717) found significant reductions in body weight, BMI and body fat percentage at 3–6 months, and a second review of 12 trials showed small-to-moderate effects across the same outcomes, with stronger effects on older adults, trials with longer interventions and populations with obesity or chronic disease [41, 42].

Collectively these findings support integrated digital strategies that combine education, self-monitoring, behavioural support and pharmacotherapy to improve multiple risk factors. However, substantial heterogeneity in intervention components limits generalisability and translation to routine ACAD care. The need for greater personalisation is echoed across trials. Future app designs should prioritise integration with primary care pathways including adherence support and prompts for medication up titration while tailoring to health literacy and cultural context.

### Conversational Agents (Chatbots)

Advances in artificial intelligence (AI) have enabled conversational agents that can simulate human dialogue via text or speech and engage in conversations with users [43]. Conversational agents are promising for behaviours underpinning atherosclerotic risk, offering scalable, personalised support across the cardiovascular disease continuum. Chatbots have been used to facilitate family communication about familial hypercholesterolemia, markedly improving cascade testing uptake [44, 45]. Accompanying qualitative evaluations report that they are preferred for communicating with younger generations [46].

Most evidence to date comes from primary prevention cohorts, and suggests small-to-moderate improvements in lifestyle behaviours [47]. A 2023 meta-analysis of 19 trials (*n* = 3,567) found that chatbot interventions produced improvements in total physical activity, steps, moderate-to-vigorous activity, fruit and vegetable intake, sleep duration, and sleep quality (SMD 0.28–0.59) across diverse age groups and settings [48]. A scoping review of 50 studies targeting cardiometabolic risk factors reported consistent gains in physical activity and weight-related behaviours, high user interest, and generally acceptable adherence, although relatively few studies assessed blood pressure, lipids, or incident cardiovascular events [49].

Drawing on data from people with established cardiovascular disease, including heart failure and atrial fibrillation, a few pilot trials have indicated potential benefits of conversational AI agents for self-management behaviours and quality of life, but robust large-scale effectiveness trials are lacking [50, 51]. Clinical endpoint trials in ACAD populations, linking agent use to sustained blood pressure and lipid control, medication adherence, and lifestyle change, remain a priority. Implementation should emphasise health-literacy–sensitive design, transparent sourcing, clinician oversight, and safe hand-offs, given risks related to privacy, bias, and plausible but incorrect content [52].

### Generative AI and Emerging Digital Tools

Large language models (LLMs) are increasingly used to draft educational content, answer patient questions, and improve readability of medical information [53].

In social-media-style assessments, chatbot responses were preferred for quality and empathy in approximately 79% of evaluations [54]. Early evaluations in primary prevention found that 84% of AI-generated answers to guideline-based questions were appropriate, although inaccuracies were present, such as prescriptive exercise recommendations for all patients and incomplete interpretation of markedly elevated LDL cholesterol without consideration of familial hypercholesterolaemia, and some content was outdated regarding newer agents [55]. In hypertension, AI responses generally aligned with guideline concepts but often lacked references and used complex language [56]. LLMs can substantially improve readability of patient materials: optimisation reduced reading grade levels of major cardiovascular (e.g., American Heart Association) and cancer websites from roughly grade 10 to grade 7, halving word counts while preserving understandability and keeping inaccuracies minimal (≤ 3.3%) [57].

Despite usability gains and promising performance in question-answering and content generation, there are currently no clinical trials demonstrating that LLM-based or generative AI interventions improve ACAD risk factors or clinical endpoints such as blood pressure, lipid levels, or sustained behaviour change.

Trust and safety require source transparency, clinician oversight, consent, and validation strategies; patients value clarity about how AI is used in their care and expect documentation of oversight [58, 59]. Technical advances through domain-specific fine-tuning must be balanced with robust data governance and environmental considerations [60]. Currently LLMs can serve as a supportive content layer, simplifying, translating, and tailoring health information to patient needs and can also help prepare individuals for shared decision making by personalising risk–benefit information and values-based choices. However, these functions must be implemented with verification against guideline-linked sources and robust guardrails to mitigate plausible inaccuracies [61].

### Digital Health Literacy

Health literacy can be conceptualised as the ability to access, understand, appraise and act on health information. Low health literacy—affecting an estimated 36–60% of adults across Australia, Europe, and the United States is associated with poorer self-management, reduced healthcare access, and higher chronic disease burden and mortality [62]. Yet many heart-health resources exceed recommended reading levels and fail to support shared decision-making: for example, online information about coronary artery calcium scoring often does not meet international standards for decision support and is written above grade 12 level; well above the recommended grade 8 level [63].

Health-literacy–sensitive co-design improves comprehension, engagement, and actionability by using plain language, chunked information, supportive visuals, and clear next steps [64]. In Australian primary care, literacy-sensitive SMS programs integrating personalised risk communication, decision aids, and tailored action plans have increased awareness and uptake of heart-health checks and facilitated shared decision making, demonstrating the value of embedding educational tools within routine workflows [64–68]. This can be supported by capacity building approaches to increase self-management skills in patient populations, such as the SMS and app approaches mentioned above.

Given the heterogeneity and variable quality of digital interventions, health-literacy–sensitive design should be considered essential for all digital tools supporting ACAD prevention and management. Pairing readable, culturally appropriate content with personalised risk information, evidence-based decision and behaviour change support, and timely integration into care pathways is likely to enhance effectiveness and reduce inequitable access to evidence-based care. However, doing this well requires capacity building to support consumer involvement beyond end-user testing, and few freely available currently exist to support this [69].

### Misinformation

Cardiovascular misinformation is common on social media and can distort risk perception, delay care, and undermine adherence to evidence-based therapies. For example, population-level studies have shown that media coverage of unsubstantiated claims can trigger widespread interruptions in medication (statin) use, with potential downstream harm [70–72]. Digital health programs, and clinical encounters must therefore proactively address misinformation by helping patients recognise anecdote-driven narratives, non-evidence-based claims, and sources with commercial conflicts, and encouraging verification against established guidelines.

As digital and AI-enabled tools become more integrated into consumer information seeking and patient communication, the risk of plausible but inaccurate content increases. Embedding counter-misinformation strategies such as source labelling, myth-versus-fact formats, prompts to consult clinicians, transparent links to guideline-verified material and co-designed interventions can support safer engagement [73]. This is especially important considering evidence from 19 trials involving 123,940 participants showing no causal relationship between statin and therapy and most adverse events listed on product labels, prompting call to review official health information sources [74]. These sources sit within the wider ecosystem of information that can also inform AI tools. Integrating these safeguards into digital educational tools and care pathways is critical for preserving adherence, maintaining trust, and reducing harm in ACAD prevention and management.

### Clinical and Implementation Implications

Digital educational strategies may support the prevention and management of ACAD, but current evidence indicates they function best as adjuncts to clinician-led care within supportive health-system infrastructures, rather than as stand-alone solutions. Their effectiveness varies by modality, population, and implementation context, and no single approach reliably improves all cardiometabolic outcomes.

Text-messaging interventions have the most consistent evidence base, particularly for supporting medication adherence and smoking cessation, with more variable and generally modest effects on blood pressure, glycaemic control, and physical activity [75, 76]. In practice, these interventions are most defensible when used to reinforce clinical recommendations, such as appointment follow-up reminders, medication-taking prompts, or brief behaviour-change cues delivered between visits, rather than as substitutes for clinical review or medication titration. Where self-monitoring is incorporated (e.g. home blood pressure or glucose monitoring), evidence suggests benefit is most likely when readings are reviewed by clinicians or linked to clear escalation pathways, although findings across trials remain mixed.

Video-based education consistently improves patient knowledge and engagement and may be particularly useful for explaining the life-course nature of atherosclerosis, treatment rationale, and preventive medications. However, effects on behaviour and clinical outcomes are heterogeneous, indicating that videos are best applied as supports for counselling, shared decision making, and self-management education, rather than as independent interventions. Peer narratives and culturally tailored content may enhance relevance and engagement for some groups, but their generalisability and scalability require further evaluation.

Conversational agents and AI-supported tools remain emerging technologies within ACAD care. Evidence to date demonstrates small improvements in lifestyle behaviours in largely primary-prevention or health-motivated populations, with limited evidence linking use to sustained cardiometabolic control or clinical outcomes. In the short term, these tools may have a role as literacy-sensitive information supports or preparation aids for consultations, provided that content sources are transparent, and clinician oversight is maintained.

Importantly, effective implementation requires more than individual access to digital tools. Professional training, workflow redesign, and organisational support are critical to ensure that educational interventions align with clinical responsibilities, do not increase clinician burden, and include clear accountability for follow-up. Without such system-level supports, digital education risks increasing patient workload and information exposure without translating into improved ACAD management.

### Future Directions

Future research should prioritise pragmatic evaluations embedded within routine care, focusing on clinically meaningful outcomes such as sustained blood pressure and lipid control, medication adherence, and appropriate treatment intensification, rather than short-term engagement or knowledge alone. Given the heterogeneity of findings to date, studies should explicitly test where, for whom, and under what conditions digital education adds value.

Rather than evaluating individual tools in isolation, future trials should examine well-defined combinations of components, such as education plus reminders plus clinician feedback, using designs that allow identification of active ingredients (e.g. tailoring, interactivity, frequency, peer voice). Alongside effectiveness, acceptability, feasibility, and clinician workload should be measured to inform real-world scalability.

Equity must be a core design consideration. This includes recruiting participants with lower health literacy, limited digital access, and higher baseline cardiovascular risk, and testing multilingual delivery, low-burden interfaces, and alternatives to smartphone-only models. Process evaluations will be essential to understand implementation barriers and unintended consequences.

For conversational agents and large language models, the field remains largely pre-trial in cardiovascular prevention. Key next steps include systematic testing, correction, and validation of AI-generated outputs in ACAD contexts, assessment of error types and safety mechanisms, and development of governance models that support safe clinical use. Only after these foundational issues are addressed should trials evaluate downstream behavioural or clinical effects.

Finally, future interventions should explicitly incorporate and test counter-misinformation strategies, including source transparency, guideline alignment, and prompts for clinician consultation, given the demonstrated impact of cardiovascular misinformation on treatment adherence and risk perception.

## Conclusion

Digital educational strategies offer scalable tools to support guideline-based prevention and management of ACAD, but current evidence indicates selective and conditional effectiveness. Future digital strategies should be health-literacy sensitive, embedded within routine care, and supported by trained clinicians and fit-for-purpose health systems. While digital education can extend reach and reinforce evidence-based prevention, it cannot substitute for clinical decision making, pharmacological management, or system-level investment. Future work should focus on pragmatic, equity-oriented evaluations that clarify how digital education can meaningfully contribute to long-term atherosclerosis prevention across populations.

## Key References

• Zaman et al. The Lancet Commission on rethinking coronary artery disease: moving from ischaemia to atheroma. The Lancet. 2025 Apr 12;405(10486):1264–312. 10.1016/S0140-6736(25)00055-8.
  - ◦ A recent Commission that aims to reduce the global burden of ACAD by shifting the focus from late-stage disease to consider the risk factors and continuum of systemic atherosclerosis.
• Laranjo et al. World Heart Federation Roadmap for Secondary Prevention of Cardiovascular Disease: 2023 Update. Global Heart. 2024 Jan 22;19(1). 10.5334/gh.1278.
  - ◦ A state-of-the-art review of secondary prevention lifestyle and pharmacological treatment of atherosclerotic cardiovascular disease. It outlines national and local roadblocks and strategies to overcome them.
• Calderon Martinez et al. Text messages as a tool to improve cardiovascular disease risk factors control: a systematic review and meta-analysis of randomized clinical trials. BMC Public Health. 2025 Apr 4;25(1):1284. 10.1186/s12889-025-21818-0.
  - ◦ A systematic review and meta-analysis (*n* = 22 studies) demonstrating blood pressure reductions and improved adherence with SMS-based interventions.
• Puljević et al. Systematic review and meta-analysis of text messaging interventions to support tobacco cessation. Tob Control. 2025 Apr 1;34(2):228–38. 10.1136/tc-2023-058323.
  - ◦ Evidence synthesis showing that SMS nearly doubles smoking cessation relative to minimal or usual care, underscoring its central role in cardiovascular prevention.
• Raffoul et al. Targeted cardiovascular risk screening through an SMS programme (Text to Detect) in general practice: a three-arm, parallel group, randomised controlled trial. The Lancet Primary Care. 2025 Nov 1;1(5). 10.1016/j.lanprc.2025.100043.
  - ◦ Large RCT demonstrating an effect of targeted SMS recall using scarcity-framed invitations and social-norm reminders about CV risk screening on rate of heart heath checks.
• Poh et al. Upskilling consumers for the digital health era: a content analysis of resources for consumer representative training. Health Literacy and Communication Open [Internet]. 2024 Oct;2(1). 10.1080/28355245.2024.2415042.
  - ◦ Content analysis of resources available at no cost to support consumer upskilling to enable their participation in the design, creation and implementation of digital health interventions.

## Data Availability

No datasets were generated or analysed during the current study.
